# 4-Amino-3-phenyl-1*H*-1,2,4-triazole-5(4*H*)-thione

**DOI:** 10.1107/S1600536808014967

**Published:** 2008-05-30

**Authors:** Yu-Yuan Zhao, Zheng Xing, Wei Dai, Guang-Fan Han

**Affiliations:** a School of Materials Science and Engineering, Jiangsu University of Science and Technology, Zhenjiang, Jiangsu 212003, People’s Republic of China

## Abstract

In the title compound, C_8_H_8_N_4_S, the planar triazole ring forms a dihedral angle of 13.7 (2)° with the phenyl ring. The crystal structure is stabilized by inter­molecular N—H⋯S hydrogen-bond inter­actions, linking the mol­ecules into chains along the *a* axis.

## Related literature

For the applications of triazole compounds, see: Xu *et al.* (2002 ▶); Jantova *et al.* (1998 ▶); Holla *et al.* (1996 ▶); Pevzner (1997 ▶). For bond-length data, see: Allen *et al.* (1987 ▶).
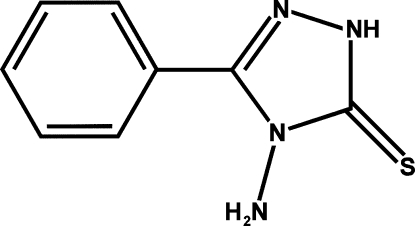


## Experimental

### 

#### Crystal data

C_8_H_8_N_4_S
*M* *_r_* = 192.25
Monoclinic, 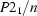

*a* = 5.5574 (4) Å
*b* = 25.2384 (3) Å
*c* = 6.6327 (4) Å
β = 104.511 (1)°
*V* = 900.63 (9) Å^3^
*Z* = 4
Mo *K*α radiation
μ = 0.31 mm^−1^
*T* = 293 (2) K
0.2 × 0.2 × 0.2 mm

#### Data collection

Rigaku Mercury2 diffractometer
Absorption correction: multi-scan (*CrystalClear*; Rigaku, 2005 ▶) *T*_min_ = 0.736, *T*_max_ = 0.939
8689 measured reflections
2134 independent reflections
1464 reflections with *I* > 2σ(*I*)
*R*_int_ = 0.062

#### Refinement

*R*[*F* ^2^ > 2σ(*F* ^2^)] = 0.067
*wR*(*F* ^2^) = 0.223
*S* = 1.12
2134 reflections
118 parameters
H-atom parameters constrained
Δρ_max_ = 0.46 e Å^−3^
Δρ_min_ = −0.44 e Å^−3^

### 

Data collection: *CrystalClear* (Rigaku, 2005 ▶); cell refinement: *CrystalClear*; data reduction: *CrystalClear*; program(s) used to solve structure: *SHELXS97* (Sheldrick, 2008 ▶); program(s) used to refine structure: *SHELXL97* (Sheldrick, 2008 ▶); molecular graphics: *SHELXTL* (Sheldrick, 2008 ▶); software used to prepare material for publication: *SHELXTL*.

## Supplementary Material

Crystal structure: contains datablocks I, global. DOI: 10.1107/S1600536808014967/rz2214sup1.cif

Structure factors: contains datablocks I. DOI: 10.1107/S1600536808014967/rz2214Isup2.hkl

Additional supplementary materials:  crystallographic information; 3D view; checkCIF report

## Figures and Tables

**Table 1:** Hydrogen-bond geometry (Å, °)

*D*—H⋯*A*	*D*—H	H⋯*A*	*D*⋯*A*	*D*—H⋯*A*
N2—H2*A*⋯S1^i^	0.86	2.46	3.310 (3)	172
N4—H4*B*⋯S1^ii^	0.89	2.67	3.506 (3)	157

Symmetry codes: (i) 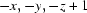; (ii) 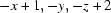.

**Fig. 1.**
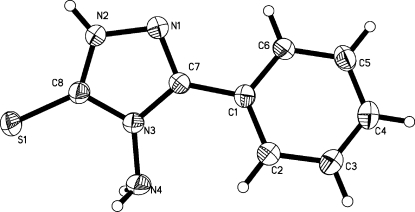
The molecular structure of the title compound with the atom-numbering scheme. Displacement ellipsoids are drawn at the 30% probability level.

**Fig. 2.**
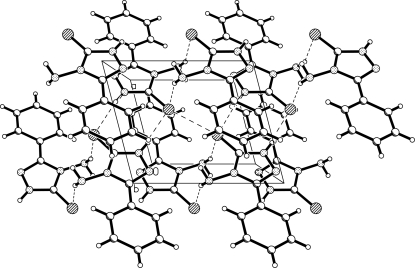
Crystal packing diagram of the title compound, viewed along the b axis. Hydrogen bonds are shown as dashed lines.

## supplementary crystallographic information

### Comment

1,2,4-Triazole and its derivatives display a broad range of biological activities, finding application as antitumour, antibacterial, antifungal and antiviral agents (Xu *et al.*, 2002; Jantova *et al.*, 1998; Holla *et al.*, 1996). Nitro derivatives of 1,2,4-triazole are also of interest as highly energetic compounds (Pevzner, 1997). In addition, studies have been carried out on the electronic structures and the thiol–thione tautomeric equilibrium of heterocyclic thione derivatives. In the search for compounds with better biological activity, the title compound was synthesized and we report its crystal structure here.

In the title compound (Fig. 1), the C—S bond length of 1.675 (3) Å is in good agreement with the mean value of 1.660 Å reported by Allen *et al.* (1987). The triazole ring is strictly planar and makes a dihedral angle of 13.7 (2)° with the phenyl ring. The crystal packing (Fig. 2) of is stabilized by intermolecular N—H···S hydrogen bonds (Table 1) linking the molecules into chains along the a axis.

### Experimental

To a solution of KOH (0.015 mol, 0.840 g) and ethyl benzoate (0.01 mol, 1.50 g) in absolute ethanol (100 ml) was added CS_2_ (0.015 mol, 0.91 ml). The mixture was diluted with absolute ethanol (50 ml) and shaken for 12 h. A suspension of the potassium salt, 98% hydrazine hydrate (0.03 mol, 15 ml) and absolute ethanol (10 ml) was refluxed with stirring for 4 h. Dilution with cold water (100 ml) and acidification with concentrated HCl precipitated a white solid. The product was then filtered and washed with cold water. Colourless crystals of the title compound suitable for X-ray diffraction analysis were obtained by slow evaporation of a solution of 100 mg in 15 ml diethylether after 3 days.

### Refinement

All H atoms were initially located in a difference Fourier map, then they were constrained to ride on their parant atoms with C—H = 0.93 Å, N—H = 0.86-0.89 Å and with *U*_iso_(H) = 1.2 U_eq_(C, N).

### Crystal data

**Table:**

C_8_H_8_N_4_S	*F*_000_ = 400
*M**_r_* = 192.25	*D*_x_ = 1.418 Mg m^−^^3^
Monoclinic, *P*2_1_/*n*	Mo *K*α radiation λ = 0.71073 Å
Hall symbol: -P 2yn	Cell parameters from 1686 reflections
*a* = 5.5574 (4) Å	θ = 3.2–27.5º
*b* = 25.2384 (3) Å	µ = 0.31 mm^−^^1^
*c* = 6.6327 (4) Å	*T* = 293 (2) K
β = 104.5110 (10)º	Block, colourless
*V* = 900.63 (9) Å^3^	0.2 × 0.2 × 0.2 mm
*Z* = 4	

### Data collection

**Table:**

Rigaku Mercury2 diffractometer	2134 independent reflections
Radiation source: fine-focus sealed tube	1464 reflections with *I* > 2σ(*I*)
Monochromator: graphite	*R*_int_ = 0.062
Detector resolution: 13.6612 pixels mm^-1^	θ_max_ = 27.9º
*T* = 293(2) K	θ_min_ = 3.2º
CCD_Profile_fitting scans	*h* = −7→7
Absorption correction: multi-scan(CrystalClear; Rigaku, 2005)	*k* = −33→32
*T*_min_ = 0.736, *T*_max_ = 0.939	*l* = −8→8
8689 measured reflections	

### Refinement

**Table:**

Refinement on *F*^2^	Secondary atom site location: difference Fourier map
Least-squares matrix: full	Hydrogen site location: inferred from neighbouring sites
*R*[*F*^2^ > 2σ(*F*^2^)] = 0.067	H-atom parameters constrained
*wR*(*F*^2^) = 0.223	*w* = 1/[σ^2^(*F*_o_^2^) + (0.1186*P*)^2^] where *P* = (*F*_o_^2^ + 2*F*_c_^2^)/3
*S* = 1.12	(Δ/σ)_max_ < 0.001
2134 reflections	Δρ_max_ = 0.46 e Å^−^^3^
118 parameters	Δρ_min_ = −0.44 e Å^−^^3^
Primary atom site location: structure-invariant direct methods	Extinction correction: none

### Special details

**Table:**

Geometry. All e.s.d.'s (except the e.s.d. in the dihedral angle between two l.s. planes) are estimated using the full covariance matrix. The cell e.s.d.'s are taken into account individually in the estimation of e.s.d.'s in distances, angles and torsion angles; correlations between e.s.d.'s in cell parameters are only used when they are defined by crystal symmetry. An approximate (isotropic) treatment of cell e.s.d.'s is used for estimating e.s.d.'s involving l.s. planes.
Refinement. Refinement of *F*^2^ against ALL reflections. The weighted *R*-factor *wR* and goodness of fit *S* are based on *F*^2^, conventional *R*-factors *R* are based on *F*, with *F* set to zero for negative *F*^2^. The threshold expression of *F*^2^ > σ(*F*^2^) is used only for calculating *R*-factors(gt) *etc*. and is not relevant to the choice of reflections for refinement. *R*-factors based on *F*^2^ are statistically about twice as large as those based on *F*, and *R*- factors based on ALL data will be even larger.

### Fractional atomic coordinates and isotropic or equivalent isotropic displacement parameters (Å^2^)

**Table:**

	*x*	*y*	*z*	*U*_iso_*/*U*_eq_	
S1	0.12788 (16)	0.01977 (3)	0.82762 (13)	0.0565 (3)	
N3	0.4947 (5)	0.08640 (10)	0.7790 (4)	0.0427 (6)	
N2	0.3057 (5)	0.05034 (10)	0.4961 (4)	0.0461 (6)	
H2A	0.2049	0.0311	0.4058	0.055*	
N1	0.4798 (5)	0.08322 (10)	0.4454 (4)	0.0478 (7)	
C1	0.7971 (6)	0.14417 (11)	0.6429 (5)	0.0413 (7)	
C7	0.5948 (5)	0.10520 (11)	0.6248 (4)	0.0405 (7)	
C6	0.9052 (6)	0.14961 (12)	0.4763 (5)	0.0465 (7)	
H6A	0.8489	0.1288	0.3581	0.056*	
C8	0.3071 (6)	0.05098 (11)	0.6963 (5)	0.0431 (7)	
N4	0.5695 (6)	0.09940 (12)	0.9922 (4)	0.0611 (8)	
H4B	0.6203	0.0702	1.0659	0.092*	
H4D	0.4409	0.1133	1.0312	0.092*	
C5	1.0959 (6)	0.18563 (13)	0.4843 (6)	0.0531 (8)	
H5A	1.1668	0.1889	0.3719	0.064*	
C4	1.1802 (7)	0.21651 (13)	0.6595 (6)	0.0566 (9)	
H4C	1.3088	0.2405	0.6658	0.068*	
C2	0.8828 (8)	0.17585 (14)	0.8169 (6)	0.0625 (10)	
H2B	0.8124	0.1732	0.9298	0.075*	
C3	1.0756 (8)	0.21171 (16)	0.8214 (6)	0.0703 (11)	
H3A	1.1330	0.2328	0.9387	0.084*	

### Atomic displacement parameters (Å^2^)

**Table:**

	*U*^11^	*U*^22^	*U*^33^	*U*^12^	*U*^13^	*U*^23^
S1	0.0563 (6)	0.0662 (6)	0.0481 (5)	−0.0193 (4)	0.0153 (4)	0.0046 (4)
N3	0.0450 (14)	0.0473 (13)	0.0346 (12)	−0.0077 (11)	0.0079 (10)	0.0004 (10)
N2	0.0478 (15)	0.0508 (14)	0.0406 (14)	−0.0148 (11)	0.0126 (11)	−0.0067 (11)
N1	0.0475 (15)	0.0538 (15)	0.0440 (14)	−0.0102 (12)	0.0153 (12)	−0.0050 (11)
C1	0.0407 (15)	0.0386 (14)	0.0446 (16)	−0.0013 (11)	0.0105 (12)	0.0017 (11)
C7	0.0432 (15)	0.0408 (14)	0.0389 (15)	−0.0019 (12)	0.0128 (12)	−0.0005 (11)
C6	0.0465 (17)	0.0545 (17)	0.0396 (15)	−0.0057 (13)	0.0130 (14)	0.0005 (12)
C8	0.0436 (17)	0.0434 (15)	0.0414 (16)	−0.0051 (12)	0.0086 (13)	0.0015 (12)
N4	0.079 (2)	0.0703 (18)	0.0332 (14)	−0.0228 (16)	0.0128 (13)	−0.0022 (13)
C5	0.0485 (18)	0.0603 (19)	0.0535 (19)	−0.0055 (15)	0.0185 (15)	0.0091 (15)
C4	0.0497 (18)	0.0535 (19)	0.068 (2)	−0.0110 (15)	0.0168 (17)	0.0027 (16)
C2	0.069 (2)	0.071 (2)	0.055 (2)	−0.0244 (18)	0.0276 (17)	−0.0158 (17)
C3	0.077 (3)	0.072 (2)	0.067 (2)	−0.031 (2)	0.027 (2)	−0.0212 (19)

### Geometric parameters (Å, °)

**Table:**

S1—C8	1.675 (3)	C6—C5	1.387 (4)
N3—C7	1.366 (4)	C6—H6A	0.9300
N3—C8	1.378 (4)	N4—H4B	0.8900
N3—N4	1.409 (3)	N4—H4D	0.8900
N2—C8	1.326 (4)	C5—C4	1.380 (5)
N2—N1	1.379 (3)	C5—H5A	0.9300
N2—H2A	0.8600	C4—C3	1.349 (5)
N1—C7	1.323 (4)	C4—H4C	0.9300
C1—C2	1.387 (4)	C2—C3	1.397 (5)
C1—C6	1.390 (4)	C2—H2B	0.9300
C1—C7	1.476 (4)	C3—H3A	0.9300
			
C7—N3—C8	109.7 (2)	N2—C8—S1	131.3 (2)
C7—N3—N4	126.7 (2)	N3—C8—S1	125.9 (2)
C8—N3—N4	123.6 (3)	N3—N4—H4B	109.3
C8—N2—N1	114.1 (2)	N3—N4—H4D	109.1
C8—N2—H2A	123.0	H4B—N4—H4D	109.5
N1—N2—H2A	123.0	C4—C5—C6	119.8 (3)
C7—N1—N2	104.1 (2)	C4—C5—H5A	120.1
C2—C1—C6	118.5 (3)	C6—C5—H5A	120.1
C2—C1—C7	123.2 (3)	C3—C4—C5	119.8 (3)
C6—C1—C7	118.3 (3)	C3—C4—H4C	120.1
N1—C7—N3	109.4 (2)	C5—C4—H4C	120.1
N1—C7—C1	122.6 (3)	C1—C2—C3	119.6 (3)
N3—C7—C1	128.0 (3)	C1—C2—H2B	120.2
C5—C6—C1	120.8 (3)	C3—C2—H2B	120.2
C5—C6—H6A	119.6	C4—C3—C2	121.5 (3)
C1—C6—H6A	119.6	C4—C3—H3A	119.3
N2—C8—N3	102.8 (2)	C2—C3—H3A	119.3

### Hydrogen-bond geometry (Å, °)

**Table:**

*D*—H···*A*	*D*—H	H···*A*	*D*···*A*	*D*—H···*A*
N2—H2A···S1^i^	0.86	2.46	3.310 (3)	172
N4—H4B···S1^ii^	0.89	2.67	3.506 (3)	157

Symmetry codes: (i) −*x*, −*y*, −*z*+1; (ii) −*x*+1, −*y*, −*z*+2.
